# Mistaken information can lead only to misguided conclusions and policies: a commentary regarding Schüz et al.’s response

**DOI:** 10.1186/s12940-023-01013-7

**Published:** 2023-09-02

**Authors:** Toshihide Tsuda, Yumiko Miyano, Eiji Yamamoto

**Affiliations:** 1 Department of Human Ecology, Graduate School of Environmental and Life Science, Okayama University, 3-1-1 Tsushima-naka, Kita-ku, Okayama, 700-8530 Japan; 2 Department of Epidemiology, Graduate School of Medicine, Dentistry and Pharmaceutical Sciences, Okayama University, Okayama, Japan; 3 Okayama University of Science, Okayama, Japan

**Keywords:** Epidemiologic methods, Thyroid cancer, Fukushima, Evidence, Nuclear accident, Radiation health effects

## Abstract

**Background:**

After reviewing selected scientific evidence, Schüz et al. made two recommendations in the 2018 International Agency for Research on Cancer (IARC) Technical Publication No. 46. Their first recommendation was against population thyroid screening after a nuclear accident, and the second was that consideration be given to offering a long-term thyroid monitoring program for higher-risk individuals (100–500 mGy or more radiation) after a nuclear accident. However, their review of the scientific evidence was inadequate and misrepresented the information from both Chernobyl and Fukushima. We wrote a review article published in Environmental Health in 2022 using the “Toolkit for detecting misused epidemiological methods.” Schüz et al. critiqued our 2022 review article in 2023; their critique, based also on their 2018 IARC Technical Publication No. 46, was so fraught with problems that we developed this response.

**Main body:**

Schüz et al. suggest that hundreds of thyroid cancer cases in children and adolescents, detected through population thyroid examinations using ultrasound echo and conducted since October 2011 in Fukushima, were not caused by the 2011 Fukushima Daiichi Nuclear Power Plant accident. Schüz et al. compared thyroid cancers in Fukushima directly with those in Chernobyl after April 1986 and listed up to five reasons to deny a causal relationship between radiation and thyroid cancers in Fukushima; however, those reasons we dismiss based on available evidence. No new scientific evidence was presented in their response to our commentary in which we pointed out that misinformation and biased scientific evidence had formed the basis of their arguments. Their published article provided erroneous information on Fukushima. The article implied overdiagnosis in adults and suggested that overdiagnosis would apply to current Fukushima cases. The IARC report did not validate the secondary confirmatory examination in the program which obscures the fact that overdiagnosis may not have occurred as much in Fukushima. The report consequently precluded the provision of important information and measures.

**Conclusion:**

Information provided in the IARC Technical Publication No. 46 was based on selected scientific evidence resulting in both public and policy-maker confusion regarding past and present nuclear accidents, especially in Japan. It should be withdrawn.

## Lack of evidence for overdiagnosis of childhood thyroid cancer

We read with interest the critique by Schüz et al. [1] to our review article [2]. We thank Schüz et al. for introducing us to an article [3] to which we did not refer in our paper. In this commentary, we explain, in our assessment, two major concerns about the process by which authors of the above two papers [1, 3] arrived at their conclusions regarding overdiagnosis of childhood thyroid cancers after the Fukushima nuclear accident.

Schüz et al. reviewed selected scientific evidence from which two recommendations were made in the International Agency for Research on Cancer (IARC) Technical Publication No. 46 “Thyroid health monitoring after nuclear accidents / IARC Expert Group on Thyroid Health Monitoring after Nuclear Accidents (2018)” [4]. The first author, Schüz was the Chair of Expert Group consisting of 14 experts. Their first recommendation was against population thyroid screening after a nuclear accident, and the second was that consideration be given to offering a long-term thyroid monitoring program for higher-risk individuals (100–500 mGy or more radiation) after a nuclear accident. We demonstrate here, that their review of the scientific evidence, however, was biased, missed many important scientific considerations, and misrepresented the information from both Chernobyl and Fukushima. We wrote a review article published in *Environmental Health in 2022* [2] using the “Toolkit for detecting misused epidemiological methods.” [5] Schüz et al. critiqued our 2022 review article in 2023 [1]. Their critique was so fraught with problems that we developed this response.

Schüz et al. explained in their response, [1] that “IARC technical publication No. 46 *Thyroid health monitoring after nuclear accidents* was a forward-looking report coordinated by IARC used scientific evidence and expertise of a large group of international scientists representing a wide spectrum of disciplines.” Although the report [4] focuses on thyroid screening using ultrasound, it does not include opinions of doctors or technicians who perform ultrasound examinations, nor of technicians who produce or engineer ultrasound equipment. The report does not present any images of ultrasound echo, or provide information on thyroid cancer or overdiagnosis in children and adolescents [2, 4]. The IARC Technical Publication No. 46 [4] is merely the opinion of a group of scientists without experts in the field of ultrasound. We believe it is important to highlight 6 major points:

## Schüz et al. started with incorrect information on Fukushima, generalized it, and applied it to Fukushima

In the article by Vaccarella et al. [3] and its references [6, 7], we identify that the authors of the papers, from among whom five, including Vaccarella, were members of the Expert Group, started with incorrect information about Fukushima. As described below, at the beginning of their article on overdiagnosis of thyroid cancer in adults [6], Vaccarella et al. stated, “In Japan’s Fukushima Prefecture, thyroid cancer incidence among screened children and adolescents was approximately 30 times as high as the national average only a few months after intensive screening programs for these age groups began in response to the 2011 nuclear accident.” [6].

The phrase “only a few months after” is incorrect; this was the case a few *years* after (specifically, 2.5 years after). The period 2.5 years was supported by evidence obtained in the interval during the first round of thyroid examinations. The second round of examinations, from April 2014 to March 2016, similarly showed an increase of 20 to 60 times the number of thyroid cancers [2], indicating that thyroid cancers had increased rapidly for 2–3 years. This evidence disproves “a slow-growing nature of thyroid cancers” as claimed in reports by the SHAMISEN (Nuclear Emergency Situations - Improvement of Medical and Health Surveillance) international experts’ consortium and the International Agency for Research on Cancer (IARC) [4, 7].

These increases, by dozens of times, in the number of thyroid cancer cases continue [8]. The overall standardized incidence ratios (SIR) in Fukushima Prefecture were 19.9 (95% confidence interval [CI]: 13.5–26.3) in the third round of screening and 27.4 (95% CI: 19.5–37.4) in the fourth round. Furthermore, a total of 51 thyroid cancer cases was identified without regular screening, including 43 cases reported by Fukushima Prefecture [9] and 8 cases reported by non-governmental organizations [9]. The former 43 cases were residents of Fukushima Prefecture under the age of 18 years at the time of the accident. These were detected in cancer registries other than the Fukushima Prefecture thyroid screening program. These 51 cases were not in any way affected by overdiagnosis because they were outside the screening program area. Their expected values were small, such that at least several dozen times more cases were still observed (SIR = 21.3; 95% CI: 15.9–28.1) despite underestimation owing to many eligible cases being missed [9].

These findings refute the overdiagnosis hypothesis in Fukushima. Despite this evidence, IARC Technical Publication No. 46 makes no mention of these findings even though the facts above were known prior to the report claiming overdiagnosis [4] as the reason for the excess was published.

In their paper on overdiagnosis in children and adolescents, Vaccarella et al. stated that “Incident thyroid cancer that occurred in children within 5 years of the Fukushima nuclear accident was considered unlikely to be due to radioiodine exposure, as tumors that were a possible consequence of radiation would have been expected to occur 5–10 years after exposure in that population.” [3] As indicated in our previous review [2], evidence in Chernobyl indicates that tumors resulting from radiation would be expected to occur 3–5 years after exposure [2, 10–12] and not 5–10 years after exposure, even before the introduction of ultrasound echo.

Experts involved in the IARC report [4], including Schüz and Vaccarella, did not initially have correct evidence regarding overdiagnosis. Those authors, including 5 members of the Expert Group, appear to have introduced the hypothesis of overdiagnosis to deal with the excess incidence of thyroid cancer in Fukushima [3, 6]. From the beginning, the authors included incorrect information on Fukushima in their paper as a possible justification for the hypothesis of overdiagnosis [3, 6, 7] as the reason for the observed excesses. Their suggestions could lead readers to believe, incorrectly, that what happened at Fukushima was a phenomenon that could not reasonably be explained by anything other than overdiagnosis.

The Expert Group then chose to generalize and apply this explanation to thyroid cancer cases in Fukushima, again, as an argument for overdiagnosis in the report [4]. To lead readers into further accepting their explanation regarding overdiagnosis, the Expert Group presented five points to counteract the effects of radiation exposure [2, 4] (In: “Thyroid cancer risks related to radiation exposure”, page 96 in the IARC report): (1) low thyroid dose in Fukushima, (2) no significant detection across different radiation areas, (3) shorter minimum latency period in Fukushima, (4) small number of thyroid cancer cases at younger ages in Fukushima, and (5) genetic pattern of Fukushima thyroid cancer cases that differed from those in Chernobyl. These points would be implied because the IARC report ignored the differences between Chernobyl and Fukushima in their comparisons with respect to exposure measurements and case counts, as well as in the age of residents [2, 4, 12]. Therefore, we refute all of these points, as mentioned in our previous papers [2, 13] and summarized in point form below:

(1) Unlike Chernobyl, exposure doses in Fukushima were not measured immediately after the accident, and we can infer that there was sufficient exposure to cause excess cases of thyroid cancer [14];

(2) Unlike Chernobyl, diagnosis was made over 2–3 years, starting from highly exposed areas, and thus the region was a confounding factor (areas screened) and caused underestimation errors [2];

(3) Unlike Chernobyl, early detection was carried out in Fukushima using ultrasound echo from the beginning, and thus the minimum latent period was shortened by a few years [2, 10–12];

(4) Unlike Chernobyl, where more than 80% of patients under 5 years of age were diagnosed, the proportion of patients in each age group in Fukushima was approximately the same [12].

(5) BRAF mutations are affected by age and were more common in Fukushima [4], and therefore this is the same reasoning as that offered in point (4) above [12].

The IARC experts provided incorrect information as opposed to evidence of thyroid cancer in children and adolescents (13) “Sect. 4.2. Epidemiology of thyroid cancer”, and “4.3. Cancer screening”, pages 30–54 in the IARC report [4]).

## Schüz et al. failed to explain how screening works and blurred the definition of overdiagnosis

The IARC report does not accurately describe the content of the screening performed in Chernobyl and Fukushima and how they differed from each other, even though the document focuses on the evaluation of population thyroid screening [4]. For example, regarding Fukushima population thyroid screening, the report mentions the existence of secondary examinations but does not verify any actual data from these examinations [4]. In Chernobyl, there was no systematic secondary examination as in Fukushima. In the IARC report [4], nearly all the information on thyroid cancer was for adults, with no clear definition of overdiagnosis provided in the report [4, 6, 15]. The report also omits the structure and methods followed to prevent overdiagnosis or false positives for thyroid cancer in Fukushima, especially in the secondary examinations [4, 8, 15].

An excess, orders of magnitude higher, in the number of thyroid cancers was observed in the second screening round in Fukushima, as well as in the first round [2, 8]. This information provides direct and compelling evidence that the observed excess thyroid cancers was caused by the nuclear accident and refutes the alternative explanation of overdiagnosis. This evidence was already well known before the 2017 meeting where the report was prepared [4]. Additionally, the report ignored evidence refuting the claim of overdiagnosis [2, 13–16]. This perpetuated incorrect information.

Implementation of the definition of overdiagnosis has wavered in research since the review article “Overdiagnosis in cancer” was written by Welch and Black in 2010 [15]. Welch and Black described cancers with possible overdiagnosis that could be studied but that did not have a specific definition, including thyroid cancer [15]. Although Welch and Black indicated that rapidly rising rates of testing and disease diagnosis in the setting of stable death rates were suggestive of overdiagnosis [15], a sharp increase in the rate of testing and diagnosis of disease could also be caused by other factors, without overdiagnosis, for example, some environmental causes and advances in cancer treatment [17, 18]. Therefore, when we observe a rapidly rising incidence, we should carefully examine each causal structure to explain the observed phenomenon.

Vaccarella et al. [3] used the inappropriate phrases, “a global increase of thyroid cancer incidence in children and adolescents” and “increased rapidly since the early 1990s in many countries and territories,” including Puerto Rico, Italy, Czech Republic, South Korea, and Turkey. Japan was not included among these countries. With the use of such phrases, Vaccarella et al. [3] generalized overdiagnosis, but there is no evidence of overdiagnosis in the increasing thyroid cancer incidence among younger people in Fukushima.

## Schüz et al. report had an adverse impact on Japanese people

We contacted *Epidemiology*, the journal in which our paper reporting on the excess thyroid cancer cases in Fukushima after the accident was published, to request the early release of our article, and issued a warning in Tokyo in October 2015 [13] to inform the Japanese people of our findings as soon as possible so that appropriate public health measures could be taken.

These actions by the Expert Group followed the unorthodox approach of seeking to cast doubt based on misinformation and led the Fukushima and Japanese governments to postpone their conclusions [19]. Despite our early warnings, the report No. 46 by the IARC Expert Group (who received funding from the Japanese government) failed to mention the above warnings [4]. The Japanese government did not incorporate these warnings into its policies. And, in Fukushima Prefecture, inconclusive talks continue endlessly, with no action being taken.

At the time of the nuclear accident in March 2011, 367,637 children and adolescents 18 years old and under at the accidents in Fukushima Prefecture were eligible for screening examinations [8]. Many of these individuals then relocated and became scattered across Japan after they reached 18 years of age. Data from Chernobyl revealed a large excess incidence of thyroid cancer after the nuclear accident, not only among children and adolescents (more than 100 times that before the accident), but also among adults (more than several times that before the accident) [11]. However, interference resulting directly from the IARC report served as a barrier to Fukushima residents being informed of important information regarding excess thyroid cancer [9]. Although excess incidence represented by dozens of times the number of thyroid cancers were harvested in the first-round examination [2, 8], dozens of times more were also observed in the second round [2, 8]. Furthermore, these substantial excesses in the incidence of thyroid cancer continued in the third and fourth rounds [8]. During this period, neither the Japanese government nor Fukushima Prefecture provided warnings or took any action. IARC [4], SHAMISEN [7] and UNSCEAR [20] may well have contributed to worsening the medical conditions of children and adolescents by having issued reports based on incorrect information since 2018, such as those regarding overdiagnosis [4, 7, 9, 20].

## Schüz et al. offered puzzling and conflicting recommendations

The IARC Technical Publication No. 46 [4] focused its attention on overdiagnosis, ignoring the differences between Fukushima and Chernobyl [2, 4], and gave little thought to the effects of the Fukushima nuclear accident.

The IARC report recommends no population thyroid screening after future nuclear accidents [4]. However, it does not recommend that the current population thyroid screening taking place in Fukushima be stopped [4]. This curious recommendation in the report became topical among the Japanese public because IARC experts such as Schüz et al. seemed to want the population thyroid screening currently underway in Fukushima to continue. If the current population thyroid screening were stopped, there would be no more overdiagnosis of thyroid cancer allegedly caused by population thyroid screening using ultrasound echo. If Fukushima Prefecture stopped population thyroid screening, it would become clear that the cause of excess incidence of several dozens of times the number of thyroid cancer cases was attributable to the nuclear accident and not from overdiagnosis. Thus, the concern among IARC experts might not have been for the health of children and adolescents [4], but rather about how to prevent their hypothesis of overdiagnosis in population thyroid screening from being refuted. This suggests to us that the recommendation against future population thyroid screening by Schüz et al. [4], but not against current population thyroid screening in Fukushima, was meant to prevent the direct observation of an excess incidence of thyroid cancer without mass screening.

## We recommend that Schüz et al. withdraw the IARC 2018 report

IARC Technical Publication No. 46 presented two recommendations [4]. Whereas the report stated, “After reviewing the scientific evidence,“ neither recommendation was supported by any direct evidence in children and adolescents [4]. Thus, these were not evidence-based recommendations [2]. By stating, without evidence, that overdiagnosis might be occurring in the Fukushima population thyroid screening program and indicating that thyroid cancer was attributable to a cause other than the nuclear power plant accident, these two recommendations in the report prevented the people of Fukushima Prefecture from receiving important information and hindered the implementation of public health measures [4, 9]. Without sufficient information regarding overdiagnosis, which had already been disproven, as well as the effects of screening, Japanese policymakers relied on the IARC report, which resulted in confusion and delayed action.

With funding from the Japanese government and power companies, in the meeting held to produce a report highlighting “overdiagnosis” based on distorted information, the IARC Expert Group developed recommendations regarding long-term strategies for large populations exposed to volatile radioiodine in the event of a possible future nuclear accident. The recommendations were not made for those affected by past nuclear accidents, including Chernobyl and Fukushima [4]. We recommend that the related published papers [3, 6] as well as the 2018 IARC report [4] be retracted.

## We highlight the usefulness of the toolkit for detecting misused epidemiological methods

The above discussion can be applied to other studies highlighting overdiagnosis on thyroid cancer by ultrasound echo [7, 15, 20], which have been disproven by many papers [2, 8, 9, 13, 16] and for which no evidence has yet been presented [2], such as in the SHAMISEN paper by Cléro et al. [7], IARC Technical Publication No. 46 [4], and the UNSCEAR 2020/2021 report [20]. These articles presented inappropriate manipulations of information that undermine science and health policy, as pointed out in our review paper [2] in which we applied the Toolkit [2].

The Toolkit was condemned by Schüz et al. as misguided, having been commissioned to conduct reviews, including biased studies, and who made recommendations for ongoing programs consistent with the wishes of the sponsors of the research in question [1, 4]. Those researchers also recommended against the further screening of residents, with or without the occurrence of a future nuclear accident [1, 4]. As for Fukushima, where excess thyroid cancers had already been detected, by not recommending the stopping of screening, Schüz et al. were able to uphold their hypothesis that these thyroid cancers were attributable to overdiagnosis. Consequently, Schüz et al. have interfered directly with the country’s current public health activities via their recommendations, which were not based on valid evidence, as if the unverifiable phenomena occurring in the country were caused by anything but the direct effects of the nuclear accident.

## Data Availability

All the data necessary to reproduce the results reported in the submitted paper are available on the websites of the Ministry of Foreign Affairs of Japan (32–34) and Fukushima Prefecture. References in this paper (8–14) are given in the text.

## Abbreviations

IARC: International Agency for Research on Cancer
SHAMISEN: Nuclear Emergency Situations - Improvement of Medical and Health Surveillance
UNSCEAR: United Nations Scientific Committee on the Effects of Atomic Radiation
SIR: Standardized Incidence Ratio
CI: Confidence Interval
